# Associations between spatial access to physical activity facilities and frequency of physical activity; how do home and workplace neighbourhoods in West Central Scotland compare?

**DOI:** 10.1186/s12942-019-0166-z

**Published:** 2019-01-29

**Authors:** Laura Macdonald

**Affiliations:** MRC/CSO Social and Public Health Sciences Unit, University of Glasgow, 200 Renfield Street, Glasgow, G2 3AX Scotland, UK

**Keywords:** GIS, Physical activity, Deprivation, Home neighbourhoods, Workplace neighbourhoods

## Abstract

**Background:**

Over a third of the Scottish population do not meet physical activity (PA) recommendations, with a greater proportion of those from disadvantaged areas not meeting recommended levels. There is a great need for detailed understanding of why some people are active while others are not. It has been established that features within home neighbourhoods are important for promoting PA, and although around 60% of time spent in exercise daily is undertaken outside the residential environment, relatively little research includes both home and workplace neighbourhood contexts. This study utilised an existing west central Scotland survey and spatial data on PA facilities to examine whether, for working adults, there are links between access to facilities, within home and workplace neighbourhoods, and frequency of PA, and whether such associations differ by socio-economic group.

**Methods:**

Using a Geographic Information System (GIS), home and workplace postcodes of a sub-sample of ‘Transport, Health and Well-being’ 2010 study respondents (n = 513) were mapped, along with public (i.e. public-sector funded) and private (i.e. private-sector funded) PA facilities (e.g. sports halls, gyms, pools etc.) within 800 m and 1600 m path/street network buffers of home and workplace postcodes. Using Analysis of Variance, associations between spatial access to PA facilities (i.e. facility counts within buffers) and self-reported PA (i.e. days being physically active in past month) were analysed. Models were run separately for access to any, public, private, and home, workplace, and home/workplace facilities. Associations were examined for all respondents, and stratified by age and income deprivation.

**Results:**

Respondents’ PA frequency was associated with spatial access to specific types of facilities near home and near home or workplace (combined). In general, PA frequency was higher where individuals lived/worked in closer proximity to private facilities and frequency lower where individuals lived/worked nearby to public facilities. Results varied by age and income deprivation sub-groups.

**Conclusion:**

This research contributes to methods exploring neighbourhood contextual influences on PA behaviour; it goes beyond a focus upon home neighbourhoods and incorporates access to workplace neighbourhood facilities. Results demonstrate the importance of examining both neighbourhood types, and such findings may feed into planning for behaviour-change interventions within both spaces.

## Background

Internationally, various physical activity (PA) recommendations have been established, which, it is believed, should be met as a minimum for individuals to reap health benefits [1, 2]. It is recommended that adults’ weekly physical activity should reach at least 150 min of moderate intensity, or 75 min of vigorous intensity, aerobic physical activity (or alternative combination of both) [2]. Regardless of the benefits of activity, it is estimated that more than half of individuals across Europe do not meet recommended activity levels, with little sign of positive change in adherence [3]. It is likely that public health could be improved considerably if populations in general, and disadvantaged groups, who are less likely to meet PA recommendations [4], in particular, increased their PA levels. Effective behaviour-change interventions require detailed understanding of the determinants of that behaviour and, therefore, it is important to investigate why some people engage in active lifestyles while others are inactive [5].

Existing studies highlight associations between features of the local neighbourhood and a number of health-related behaviours [6–9]. In the past 15 or so years, studies of environmental or ‘built environment’ influences on the determinants of PA have come to the fore [10]; such research has been supported by the growth in recognition, and the popularity, of Geographic Information System (GIS) software. Previous analyses of associations between the built environment and subsequent PA [such as walking or moderate-to-vigorous physical activity (MVPA)], has included environmental characteristics such as street connectivity, residential density, net retail, and land use, greenery, presence of cycle paths, sidewalk/pavement availability, spatial access to recreational or exercise facilities etc. Although findings vary, many built environment features have been found to be positively associated with PA [10–14].

One possible, and perhaps relatively straightforward, intervention in the built environment is improving the provision of PA facilities such as spatial access to gyms, sports halls, swimming pools etc. The assumption is that if facilities are readily accessible, PA may become more likely due to a reduction in travel time, or where other exercisers are seen around facilities, PA becomes ‘normalised’ [15]. It is not surprising therefore that there has been much interest in investigating links between PA and proximity to PA facilities (see for example [15–22]). Spatial access to PA facilities shows both spatial and social inequality; results are mixed with existing research showing decreasing PA facility density with increasing deprivation in England [23], or poorer spatial access within deprived areas in Wales [24] and Scotland [25] to private facilities specifically. It is important to explore whether such inequality in provision is related to inequality in PA levels. Furthermore, relatively little research investigated how access to different PA facility types, such as public or private, may contrast in associations with PA frequency. Two US studies observed significant positive relationships between exercise and home neighbourhood private facilities, but not public facilities [15, 26], which the authors hypothesised may be due to private facilities greater appeal to users in terms of quality, attractiveness or a wider availability of features.

Much existing research focuses on the PA environment or ‘activity space’ around home locations [15–21]. However, previous research in England found that 60% of light/MVPA occurred *outside* residential neighbourhoods [27]. Therefore other spaces, such as the ‘workplace’ neighbourhood, where individuals may spend much of their day, could play a significant role in health behaviours [28, 29]. Currently, 73% of 16–64 year-olds in the UK are in employment [30], with similar proportions across the European Union (71.1% for 20–64 year olds) [31]. The workplace context therefore provides potential for engaging in exercise for a large majority of that population. Surprisingly few studies to date have looked at environmental supports for PA in proximity to the workplace. Those that have done so have mainly been US-based [13, 28, 29, 32, 33]. Within these studies associations were seen between higher PA facility density within workplace areas and higher walking rates [28], and lower body mass [29], and variations seen between home and workplace areas for built environment correlates of MVPA [13]. A recent study based in the US found that including both home and work neighbourhood walkability strengthened positive associations with MVPA in the work neighbourhood for females (home neighbourhood MVPA not included within PA outcomes) [34]. Other US based research found home neighbourhoods’ and workplace neighbourhoods’ bicycle and recreation facilities [32], and walkability [33] to be positively associated with PA, however both papers used respondent perceptions of neighbourhood features only which may not correspond accurately to objective measures [35]. A recent study in central Japan showed an association between presence of home neighbourhood sports facilities and likelihood of habitual exercise, i.e. exercising 3 or more times a week (in men only); there were no significant associations between workplace neighbourhood sports facilities and exercise [36].

There is a dearth of research exploring both home and workplace environmental influences on adult PA behaviour in the UK. In addressing this gap, this study draws on data from the West of Scotland, UK. This region shows low proportions of adults meeting recommended PA levels (45% of men and 33% of women [37]) in particular for those in the most deprived households [38], in addition to poor population health [39], adding to the urgency and saliency of the study.

This study aims to contribute to methodology for quantifying potential environmental supports for PA (i.e. through PA facility access) in both home and workplace neighbourhoods. The study provides a novel application of this methodology to the context of the UK as this topic appears under-researched within UK based studies. The research questions are:-

1. Is PA frequency associated with spatial access to PA facilities in home and/or workplace neighbourhoods?
2. Do associations vary by:
  - PA facility proximity
  - PA facility location
  - PA facility type
  - Age
  - Sex
  - Income deprivation

## Methods

### Study data

Data were from the ‘Transport, Housing and Well-being Study’ THAW [40]—a cross-sectional study which drew respondents (adults aged 18 to 95 years old) from West Central Scotland. The postal questionnaire included questions on mental and physical health, housing, neighbourhood, transport, employment, and health behaviours (for further information see [41–43]). 1094 respondents were categorised as currently working in Scotland, however, 41 did not provide age or PA data, and 540 did not provide a full/accurate workplace postcode so were excluded. The final sample included 513 people who reported their employment status as full-time/part-time, had accurate home and workplace postcodes, and had no missing data for age, sex, or PA. *Missing data*: Those included and excluded from analysis did not vary greatly in terms of age, sex or PA days (included: 53% female, mean age: 44.9 years, mean PA days: 9.8; excluded 54% female, mean age: 46.9 years, mean PA days: 10).

### Linking home location to income deprivation

Respondent postcodes were linked to ‘data zone’ codes [small area statistical geography formed from groups of Census output areas, and comprising of between 500 to 1000 residents [44]] and then to ‘Scottish Index of Multiple Deprivation (SIMD) 2009’ ‘Income domain’ scores, which are based on numbers of claimants for various welfare benefits [45] (look up files obtained from [46]). SIMD scores were divided into tertiles (T1 = lowest scores/least income deprived, T3 = highest scores/most income deprived). This area-level measure of income deprivation is referred to in this paper as income deprivation.

### Physical activity measure

PA frequency was extracted from the question: *“On how many days in an average month do you do any sport or physical exercise (e.g. dancing or brisk walking) that makes you out of breath and sweat, and that you do for more than 20* *min at a time?”*. Similar question wording has been used previously in THAW 1997 [47] and the West of Scotland Twenty-07 Study [48]. This wording is appropriate for capturing information on the number of days undertaking at least moderate intensity activity [49].

### Mapping respondent postcodes

All geo-referencing was performed using ArcGIS version 10.3. Home and workplace postcodes were geocoded to X and Y coordinates via the Office for National Statistics Postcode Directory for August 2010 [50] (which contains British National Grid coordinates for address-weighted unit postcode centroids), and two shape files (i.e. geospatial file storage format) were created—one file for home location points and another for workplace location points.

### Mapping physical activity facilities

A list of PA facilities across Scotland for 2009 was obtained from Sportscotland (the national agency for sport in Scotland) [51]. The list included both ‘permanent’ facilities (e.g. football pitches, hockey pitches, tennis courts, swimming pools, gyms etc.), and also other facilities used intermittently for exercise (e.g. sports halls within schools and community centres). The dataset included precise geo-coordinates, full postal addresses and whether the PA facility was public (i.e. public sector funded facilities subsidised by national/local government) or private (i.e. private sector funded facility set up by a private company for members only). PA facilities were mapped using these geo-coordinates.

### Additional spatial data

An ‘Integrated Transport Network Layer (ITN) (and path network)’ for Scotland (2011), i.e. an Ordnance Survey dataset containing transport infrastructure information, and Scottish data zone boundaries and geometric centroids, were obtained [52, 53].

### Spatial analysis—creating network buffers

All spatial analysis was performed within ArcGIS v10.3. 800 m and 1600 m ‘network’ buffer polygons were created around respondents’ home and workplace postcode centroids. These polygons are calculated by tracing the defined distance from a point along road/path networks in all possible directions, with adjacent route ends joined by connecting lines to form an enclosed area. These buffer extents are commonly used to capture PA environment as they could be described as ‘walkable distances’ [13, 16, 19, 29]. Two buffer extents were chosen to allow for sensitivity analysis to explore differences in the relationship between PA level and PA facility access (as defined by the two buffer distances). At an average walking speed of 5 km-per-hour [54], 800 m would take around 10 min to walk.

### Spatial analysis—quantifying ‘access to facilities’

To calculate the key explanatory variable ‘access to facilities’ the join tool was used to spatially locate facilities within respondent home and workplace neighbourhood buffers. Facilities sum was calculated for every respondent home buffer and workplace buffer, for all facilities, for public facilities and for private facilities. Counts of facilities for each buffer were ordinally categorised into three levels—none, one, and two or more.

### Statistical analysis

All statistical analysis was undertaken using IBM SPSS Statistics version 21. General Linear Models for Analysis of Variance (ANOVA) were used to assess associations between the quantitative PA outcome (i.e. numbers of physically active days per month (PA frequency)) and access to PA facilities (i.e. none, one, two or more facilities). Various models were run (n = 18) using all combinations of PA facility proximity, location and type to define access to facilities. Home neighbourhood models included separate analysis for PA facility proximity and PA facility type e.g. any, public, and private facilities within 800 m and 1600 m buffers. This was repeated for workplace neighbourhoods and home/workplace neighbourhoods. Models were adjusted for age, sex and income deprivation (see Table 1 for a summary of the variables and categorisation).

**Table 1:** Summary of variables

Variable (type)	Categories
PA frequency (dependent)	Ranging from 0 to 31 days
PA facility access (independent/variable of interest)	None, one, two or more
PA facility proximity (independent/variable of interest)	800 m buffer, 1600 m buffer
PA facility location (independent/variable of interest)	Home neighbourhood, Work neighbourhood, Home/workplace neighbourhood
PA facility type (independent/variable of interest)	Any, public, private
Age (independent/potential confounder)	19–39 years old, 40–49 years old, 50 + years old
Sex (independent/potential confounder)	Males, females
Income deprivation tertiles (independent/potential confounder)	Lowest, middling, highest

### Sub-group analyses

In all models above interactions were included between the primary explanatory variables (access to facilities) and each of the potential confounders (sex, age and income deprivation). Significant interactions were found between age and public facility access (within 1600 m workplace buffers), and between income deprivation and private facility access (within 800 m workplace buffers). Associations between public facility access (within 1600 m workplace buffers) and PA frequency, were explored separately for the three age groups, while associations between private facility access (within 800 m workplace buffers) were explored separately for the three levels of income deprivation. Age group models were adjusted for sex and income deprivation, and income deprivation group models adjusted for age and sex. Where significant differences in PA frequency were found across levels of access to facilities, post-hoc testing was carried out using Bonferroni correction (reducing the likelihood of Type 1 errors [55]), allowing pairwise comparisons between levels of access to facilities.

## Results

The sample included 293 female and 220 male working adults, with a mean age of 44.9 years (range 19–74 years) (19–39 (n = 167), 40–49 (n = 150), 50 + (n = 196)). The majority had at least one active day in the past month (83.8%), with a mean of 9.8 active days (range 0–31 days). Table 2 shows differences in average number of PA days for the various sex, age and income deprivation sub-groups. There were no sex or age differences in PA days, but for income deprivation tertiles (T), those within the lowest income deprivation group displayed the lowest mean PA frequency (p = 0.04).

**Table 2:** Physical activity frequency (monthly) descriptives (min–max = 0–31 for all)

	N	Mean (median)	SD	ANOVA F-value, p-value
All	513	9.8 (8.0)	8.29	
Sex				
Males	220	10.5 (8.0)	8.61	
Females	293	9.3 (8.0)	8.01	F = 2.674, p = 0.103
Age				
19–39	167	10.1 (8.0)	7.71	
40–49	150	9.8 (8.0)	8.50	
50 +	196	9.5 (8.0)	8.62	F = 0.206, p = 0.814
Income deprivation				
1 (higher)	180	10.0 (8.0)	7.65	
2	163	10.9 (8.0)	9.25	
3 (lower)	170	8.6 (7.0)	7.84	*F* = *3.120, p* = *0.04*

### Comparing home and workplace access to facilities

Of all respondents, 85.8% lived, and 88.7% worked, within 1600 m of at least one PA facility of any type. In comparison to 1600 m home buffers, 1600 m workplace buffers had significantly higher mean number of facilities of any type (home: 3.3 and workplace: 4.5, p = 0.01), and private facilities (home: 1.2, workplace: 2.0, p = 0.01). When comparing PA facility access between the least (T1) and most (T3) income deprived areas, those within T1 had better access at home to any, public or private facilities; 94% had at least one PA facility of any type within 1600 m of home with a mean of 3.6 (compared to 82.2% and a mean of 2.7 for less deprived areas). For workplace areas, 90% of the individuals from T3 had a PA facility within 1600 m, compared to 86.5% of those within T1. Private facilities were also more accessible in the least deprived workplace areas.

### Access to PA facilities and PA days

Table 3 displays associations between PA days (i.e. number of days physically active in an average month) and access to facilities (none, one, two or more) of any, public and private facilities within 1600 m of home, and workplace locations.

**Table 3:** Mean PA days^a^ by access to facilities within home and workplace buffers

	PA days Mean	S.E.	(F-value, p-Value)
PA facility access within 1600 m of home			
Any			
None (73)	10.0	0.99	
One (66)	8.8	1.02	
Two or more (374)	10.1	0.43	(F = 0.8, p = 0.46)
Public			
None (115)	10.5	0.79	
One (138)	10.0	0.71	
Two or more (260)	9.6	0.52	(F = 0.4, p = 0.66)
Private			
None (253)	8.9	0.52	
One (102)	10.7	0.82	
Two or more (158)	11.1	0.67	*(F* = *3.8, p* = *0.02)*
PA facility access within 1600 m of workplace			
Any			
None (58)	8.8	1.09	
One (46)	9.2	1.22	
Two or more (409)	10.2	0.41	(F = 0.9, p = 0.40)
Public			
None (118)	9.6	0.76	
One (133)	10.1	0.72	
Two or more (262)	10.0	0.52	(F = 0.2, p = 0.84)
Private			
None (186)	9.3	0.61	
One (92)	9.2	0.87	
Two or more (235)	10.8	0.54	(F = 2.1, p = 0.12)

^a^Adjusted for age, sex and income deprivation

### Home neighbourhoods

There was a significant association between access to private facilities within 1600 m of home and PA frequency (p = 0.02); specifically there appeared to be a slight cumulative benefit to exercise from access to one private facility (mean PA: 8.9) to access to two or more private facilities (mean PA: 11.1) within 1600 m of home (p = 0.04).

For PA facility access within 800 m buffers (results not tabulated), those living within 800 m of two or more public facilities showed significantly lower PA days (6.2) than those with none (10.3) within 800 m home buffers (p = 0.01).

### Workplace neighbourhoods

There were no significant associations between PA days and access to any, public, or private facilities within 800 m or 1600 m of workplace

### Home and workplace neighbourhoods combined

For home and workplace neighbourhoods combined there were no significant associations between PA frequency and the three-category PA facility access variables (i.e. none, one, or two or more) therefore additional analysis was undertaken with two-category PA facility access variables (i.e. none, or one or more). Respondents showed increased PA days with one or more private facilities within 800 m of home/workplace (none: 9.0 PA days, one or more: 10.8 PA days, p = 0.03) but decreased PA days with one or more public facilities within 800 m (none: 10.8 PA days, one or more: 9.2 PA days, p = 0.03) (no significant findings for 1600 m buffers) (tables not shown).

### Goodness of fit

To compare the goodness of fit of statistical models relative to each other (i.e. home facilities model to home/workplace facilities model) Akaike’s Information Criterion was used (AIC) [56]. Compared to the home models, the home/workplace models displayed lower AIC values suggesting better model fit, and that including both home neighbourhood and workplace neighbourhood facilities within ‘access to facilities’ better described the differences in PA between groups.

### Age and income deprivation sub-groups

Similar to the home/workplace combined analysis, sub-group analysis was undertaken with twofold PA facility access variables (i.e. none, or one or more). Those within the youngest age group showed increased PA days where one or more public facilities were located within 1600 m of work (none: 7.7 PA days, one or more: 11.3 PA days, p = 0.01), while those within the lowest income deprived group showed greater PA where one or more private facilities were located within 800 m of work (none: 9.3 PA days, one or more: 11.0 PA days, p = 0.05).

## Discussion

This research contributes to methods exploring the neighbourhood context in influencing PA behaviour, going beyond a focus solely upon the home neighbourhood by incorporating access to facilities within workplace neighbourhoods. We looked at whether there is an association between frequency of PA and spatial access to PA facilities within either home and/or workplace neighbourhoods for working adults. The study also highlights the importance of stratifying analysis by PA facility type, given that little existing research investigated how different facility types affected the association between access and PA frequency. We found that associations varied depending on spatial access to facilities in home neighbourhood, workplace neighbourhood, and to public or private facilities. Those living closer, or living/working closer, to private facilities, exercised more frequently than those with nearby access to public facilities.

### Comparison with existing literature

A previous US Study found that areas around home contained more fitness facilities than non-home areas [57], while we found that workplace neighbourhoods had more facilities. Variation in proportions of open space, building density etc. may influence the categories of facilities that are situated within an area. For THAW respondents, workplace postcodes were more likely than home postcodes to be located within built-up urban centres (66% of workplace postcodes located in large urban areas compared to 56% of home postcodes). Both THAW home and workplace neighbourhoods appeared to play a role in influencing health behaviours, as found by previous US-based studies looking at access to recreational facilities and BMI [29], and walkability measures and MVPA [13, 34]. Two of these US studies found that built environment features of the home neighbourhoods were more influential for the particular health outcomes than workplace neighbourhoods [13, 29], which agreed with findings for the non-stratified analysis within our study, while Marquet et al.’s [34] findings suggested that the combination of home and workplace neighbourhood walkability was more powerful in explaining workplace environment PA than the workplace neighbourhood alone. The authors argued the home environment could potentially influence PA behaviours and habits that are then extended to the work environment. Research undertaken in Aichi, Japan, found an association between PA and home neighbourhood sports facility access (for males only) which did not exist for work neighbourhood facility access; the authors hypothesised that the lack of association could be partly due to limited time for exercise during the working day [36].

Our study found no significant association between frequency of PA and level of access to facilities of any type. The majority of our sample lived or worked within 1600 m of a PA facility of some type (85.6% and 88.7% respectively), perhaps due to being highly urban. Therefore, access to facilities by type did not vary greatly. This limited variability may contribute to the lack of association between exercise and level of access to facilities of any type around home and/or workplace. There appeared to be a greater benefit to exercise frequency for those living closer to private facilities than public within our analysis. Relatively few studies compared the influence of public or private facilities access on exercise, although some compared ‘free’ public facilities and ‘pay’ facilities. Two US studies observed a significant positive relationship between exercise frequency and private facilities around homes, but not for public facilities [15, 26], which agrees with our findings. Unlike the two US studies, this study demonstrated that PA days were lower when public facilities were proximal. The positive association between frequency of PA and level of access to private facilities, and the negative association between frequency of PA and level of access to public facilities, within our study, could be due to additional factors not included here, such as ‘quality’ or ‘attractiveness’ [26]. Although not all public facilities in the study were free, it could be assumed fees are lower as usage fees are subsidised. This may have been true when the THAW data was collected but more recently there has been an increase in the popularity of ‘cheaper’ private chain gyms in the UK such as ‘PureGym’ [58]. These private facilities are increasingly lower budget, offer flexible contracts or ‘pay as you go’, and provide an array of equipment and fitness classes. Public facilities on the other hand may be perceived to be, or may actually be, lower in quality. Within our study, a large number of the public PA facility sites were team sport pitches (e.g. football pitches), which made up two thirds of the public facilities compared to one fifth of private facilities. Previous research demonstrated that the presence of football pitches had a negative impact on exercise behaviour [20]. Other factors may be at play which make PA resources inaccessible to respondents, e.g. previous qualitative work showed that some people did not use proximal green space due to perceptions of it being unsafe or not ‘for them’ [59]. Future qualitative work could explore the social environment and perceived crime safety around facilities such as football pitches.

In terms of sub-group differences, only the youngest group and the lowest income deprivation group displayed associations between PA facility access and PA frequency. The lowest income deprivation sub-group appeared to gain the most exercise benefit from proximal private facilities compared to the other income deprivation groups. Previous research demonstrated that access to neighbourhood PA resources influenced exercise habits of the socially disadvantaged groups to a greater extent than the PA of their more advantaged counterparts [26, 60, 61]; within our study this appeared to be true for workplace neighbourhood only. Other research looking at the size of study participants’ self-defined neighbourhoods, found that compared to the less educated, more educated individuals defined their neighbourhood to be *larger* in size [11]. The authors maintained that the better educated experienced “greater mobility in terms of frequency and distance, and at the same time live in places with greater access to urban opportunities such as services, transportation and social activities” ([11], p. 15). Perhaps in our study few associations were found between access to local facilities and exercise because high and middling income deprivation respondents tend to travel further to access PA resources than their lower income deprivation counterparts. Indeed previous research comparing walkability scores across urban Scotland found more deprived neighbourhoods to be more ‘walkable’ than less deprived areas, with poorer areas showing better connected street networks [62]. This could facilitate more direct access to local facilities.

Although the focus of this study is upon Scotland, the findings are relevant to, and method replicable within, research based beyond the UK. The prevalence of physical inactivity in other developed Western nations, including the rest of the UK and Europe, is similar to Scotland [63]; with reported declines in PA and increases in obesity [3, 64]. The results of this current study can feed into discussions on settings based research within other developed countries where existing equivalent studies may be few. Indeed with spatial data, for use within GIS, more readily available across Europe [65] there is potential and value to exploring neighbourhood supports for PA within different countries and contexts.

### Strengths and weaknesses

This study displayed a number of strengths; a strong feature being that both the home and workplace locations were examined when looking at PA facility access and exercise outcomes. It has been maintained that true associations between PA facility access and PA could be underestimated by restricting research to features of the home context [27]. A number of papers studied home neighbourhoods only [15, 18–20, 66, 67], while our study went beyond this by including PA facility access data for home and workplace areas, observing significant associations with exercise for both. We used objectively measured spatial access to facilities, while previous work made use of perceived access to neighbourhood features only [32, 33]. Associations between perceived access and PA have been previously demonstrated but findings must be interpreted with caution as the accuracy of self-reported access to neighbourhood features may vary by individual factors, e.g. less active people may overestimate distance to destinations than their more active counterparts [68]. We also utilised road/path network buffers; network distance measures are more precise taking into consideration route barriers [69], and including both roads and paths in the network provided more realistic models of potential pedestrian movement. A further strength included stratifying analyses by PA facility type and sub-group; few studies compare the influence of access to specific exercise facilities (such as public or private) on exercise behaviour [20].

In terms of limitations, this study was cross-sectional, hence we cannot assume that associations between key variables are causal. For example, we cannot say from this study whether any associations were due to more active respondents ‘self-selecting’ to live or work in areas with better access to sports facilities, as this data was not available. Previous work highlighted the significance of considering ‘residential self-selection’ but found that it did not singularly account for links between features of the built environment and PA [70, 71]. The overall sample size (n = 513) was large enough for meaningful analysis but many individuals were excluded due to a lack of workplace postcode. It must be considered that missing data could be biased towards certain groups and these groups may be under-represented, however we have no reason to believe that lack of recall, or error in reporting, of a workplace postcode would be more or less likely within a particular socio-economic group, and included and excluded groups did not differ greatly in terms of age, sex and PA frequency. Furthermore, PA facility access measures within this study could be classed as limited, as only spatial proximity was assessed, and being physically close to a PA facility does not necessarily equate to use. Use of a PA facility will likely depend on not only spatial proximity but interactions with other factors such as opening hours, scheduling of exercise classes, fees, etc. Nonetheless previous research did find that better spatial access to a PA facility was linked to higher levels of usage of that particular facility [18]. No significant link between access to exercise facilities and MVPA was seen, but PA facility usage patterning led authors to maintain that access did augment individuals’ efforts to meet recommended exercise levels by providing ‘opportunities’ [18]. Information on which specific exercise resources respondents used would enhance our analysis but this data was not collected.

Beyond built ‘formal’ PA facilities there are additional aspects of the built environment, not included in the current study, which could influence PA behaviour such as walking, cycling or other unstructured activity. Higher rates of walking were found amongst those living [9, 12, 34] or working [34] within highly ‘walkable’ neighbourhoods (i.e. with higher street connectivity, residential density, mixed land use etc.) while greater cycling and walking rates have been linked to living in areas with better access to green space and bicycle facilities [11]. Further work could include a greater range of home and workplace neighbourhood built environment variables potentially related to PA, or include data on presence of fitness equipment within homes or workplaces; fitness equipment access within the workplace has been linked to higher cardio-respiratory fitness (in women) [72]. The current study examined associations between PA facility access and overall PA behaviour, however Giles-Corti et al. [18] argued that prediction of PA will be improved where ‘behaviour-specific’ environmental features are associated with ‘context-specific’ behaviours. Detailed PA behaviour data was unavailable within the THAW study however it is likely that a range of sport and physical exercise activities were undertaken by respondents. Investigation of specific environmental factors encouraging specific activities e.g. playing competitive sports, using a gym, or walking for leisure etc. [73] could also be included in future research.

## Conclusion

The study contributes to the limited research on associations between built environment features and exercise frequency within the context of west central Scotland and contributes to methods exploring the neighbourhood context in influencing PA behaviour, going beyond a focus solely upon the home neighbourhood by incorporating access to facilities within workplace neighbourhoods where individuals may spend much of their day. Links between access to PA facilities and exercise differed when looking at facilities at home or at workplace neighbourhoods, and findings varied by type of PA facility. Respondents had relatively easy access to exercise facilities within home and workplace neighbourhoods however the PA facility type may be the key factor in influencing exercise levels; private, rather than public, facilities appeared to positively influence PA. The findings in this study suggest that physical activity supports within both home and workplace neighbourhood context influence workers’ health behaviours; these findings may feed into planning for behaviour-change interventions within both spaces. Future work could explore how service charges and the quality and attractiveness of services within public and private facilities differ, and how home and workplace environments interact to influence health behaviours.

## Abbreviations

PA: physical activity
GIS: geographic information system
THAW: transport, housing and well-being study
ANOVA: analysis of variance
MVPA: moderate-to-vigorous physical activity
SIMD: Scottish index of multiple deprivation
p: probability value
